# Democratizing cardiac imaging with an automated magnetic resonance exam

**DOI:** 10.21203/rs.3.rs-6857034/v1

**Published:** 2025-07-18

**Authors:** Danielle Kara, Ashmita Deb, Hoa Le, Daniel Wee, Makiya Nakashima, Mohsen Darayi, Tassia Ribeiro Salles Moura, Mary Robakowski, Heather Kohut, Madihah Kazim, Yuncong Mao, Lifu Deng, Fayez Kanj, Yea-Lyn Pak, Zackary Goff, Angel Houston, Kathy Kohut, Dingheng Mai, Thomas Garrett, Emma Wexler, Jeffrey Mlakar, Andrew Dupuis, Yiling Fan, Masafumi Sugawara, Ellen Roche, Mark Griswold, Richard Grimm, Samir Kapadia, Lars Svensson, Oussama Wazni, Stephen Jones, Hiroshi Nakagawa, H.W. Wilson Tang, Michael Bolen, Daniel Lockwood, Debkalpa Goswami, Deborah Kwon, David Chen, Christopher Nguyen

**Affiliations:** Cleveland Clinic; Cleveland Clinic; Cleveland Clinic; Cleveland Clinic; Cleveland Clinic; Cleveland Clinic; Cleveland Clinic; Cleveland Clinic; Cleveland Clinic; Cleveland Clinic; Cleveland Clinic; Cleveland Clinic; Cleveland Clinic; Cleveland Clinic; Cleveland Clinic; Cleveland Clinic; Cleveland Clinic; Cleveland Clinic; Cleveland Clinic; Cleveland Clinic; Case Western Reserve University; Case Western Reserve University; Massachusetts Institute of Technology; Cleveland Clinic; Massachusetts Institute of Technology; Case Western Reserve University; Cleveland Clinic; Cleveland Clinic; Cleveland Clinic; Cleveland Clinic; Imaging Sciences, Imaging Institute, Cleveland Clinic, Cleveland, OH; Cleveland Clinic; Cleveland Clinic; Cleveland Clinic; Cleveland Clinic; Cleveland Clinic; Cleveland Clinic; Cleveland Clinic; Cleveland Clinic

## Abstract

Advanced imaging of the heart, including cardiovascular magnetic resonance imaging (CMR), has revolutionized the diagnosis and prognosis for cardiovascular disease^1–3^. For the past 40 years, CMR has primarily relied on the acquisition of numerous breath-held 2D images resulting in complex scanner operation, patient discomfort, long scan durations, and cumbersome image interpretation^4,5^. These limitations constrain CMR use to major academic hospital systems and severely limit patient access to CMR, which makes up < 1% of total cardiovascular imaging despite being represented in two thirds of all AHA/ACC guidelines^6,7^. By leveraging advanced multidimensional physics and artificial intelligence, we overcome these challenges by developing a 30-minute end-to-end automated CMR exam (AutoCMR) that delivers 4D anatomical, functional, and tissue characterization of the whole heart in a single click without breath-holds. AutoCMR was rigorously validated in three cohorts: preclinical large animals, patients scanned in an academic hospital setting with over 40 years of CMR experience, and patients scanned in a community health center with no prior CMR experience. While providing simplified CMR acquisition and automated analysis, we demonstrated that AutoCMR was not significantly different than conventional CMR in imaging biomarkers and human interpretation. With its 4D whole thoracic coverage, we further showcased that AutoCMR can enable next generation patient analytics including personalized digital twins, 3D printing, virtual reality, and automated clinical structured summaries. Due to its inherent scalability, we anticipate AutoCMR will promote the democratization of CMR, increasing patient access for all including underserved health communities, while enabling powerful downstream cutting-edge technologies aimed at personalized medicine.

## Introduction

Advanced imaging of the heart has revolutionized the diagnosis and prognosis of cardiovascular disease, which represents the leading cause of death in the world^8,9^. However, access to advanced cardiovascular imaging varies significantly based on geographical location and healthcare infrastructure^10^. One key advanced cardiovascular imaging modality is cardiac magnetic resonance (CMR), which particularly faces challenges with scarce access^7^ despite being represented in two thirds of ACC/AHA guidelines^6^.

CMR is an imaging modality integral to the diagnosis, prognosis, and treatment of cardiovascular diseases^11–14^, providing morphology, function, tissue characterization, and angiography in a single study. Key characteristics relevant to its flexibility are a combination of high spatial resolution (1–2mm), high temporal resolution (> 25fps), and ability to interrogate myocardial tissue characteristics without ionizing radiation. Despite these advantages and the increasing demand for advanced cardiac imaging^15^, both growth and overall volume of CMR is 20–100x lower compared to the other advanced cardiac imaging modalities^16^.

The way the CMR exam is acquired has not fundamentally changed over the past 40 years, ultimately limiting access and growth. A conventional CMR study consists of dozens of 2D images acquired as slices through the heart, with cardiac gating using an ECG signal to address the heart’s motion^4^. Capturing these images without respiratory motion artifacts requires repeated breath holds, resulting in long and arduous exams for many patients with heart disease who already have difficulty breathing. Every acquired 2D image not only increases total scan time but also taxes patient stamina. Although CMR is capable of flexible visualization of all cardiac anatomy, the constraints of this breath-held 2D imaging approach necessitate a narrow imaging protocol focusing on the left ventricle (LV) and sacrificing comprehensive visualization of the atria, right ventricle (RV), and aorta^17^. Images must be acquired at carefully planned, patient-specific slice locations and orientations to provide the desired clinical information. Technologists must adapt in real-time to patient breath-hold capability, poor cardiac gating, and susceptibility artifacts arising, for example, from cardiac implants and blood flow. As a result, conventional CMR is a technically involved and slow imaging modality which requires a highly specialized CMR-specific technologist who has deep understanding of cardiac anatomy, MRI physics, and individual patient capabilities to achieve a diagnostically useful study^18^. Despite the limited exam design focusing on the LV, CMR scan-time remains long and variable^19^. Therefore, in addition to the need for a specialized technologist, challenging scheduling logistics and reduced economic viability compared to shorter, more easily acquired head and extremity MRI limit the integration of CMR on mixed use MR systems and largely restrict CMR implementation outside of academic hospitals^17^.

Given these constraints, it is increasingly recognized that there is a need to simplify the CMR study in order to make it more accessible^17^. Developments in highly accelerated imaging have demonstrated widespread success in reducing acquisition times and improving patient comfort by shortening breath-hold time. However, the inherent limitation of 2D imaging remains with the overall number of breath-holds and total scan time largely unchanged. More importantly, a high bar of anatomic knowledge is still necessary to accurately place slice locations. Automated scanning using artificial intelligence (AI) has also been proposed to prescribe slices^20^. There has been wide commercial push for this with many major vendors releasing some form of automated scanning or scanning aid, demonstrating success in shortening scan times^21,22^. However, such systems still require an experienced technologist if the automation fails, and the standard exam still suffers from the need for repeated breath-holds and the limited spatial coverage inherent of 2D scanning. Increasing access to CMR outside of the academic medical center requires a paradigm shift in how CMR is currently performed. Specifically, CMR needs to move away from the manually and expertly prescribed, breath-held 2D imaging exam and towards promoting a simplified exam with a consistent scan time.

Free-breathing 3D cine or delayed enhancement (DE) imaging have been proposed to improve cardiac coverage, reduce total acquisition time, and improve patient comfort with free-breathing acquisitions^23–27^. However, many of these methods require careful optimization of cardiac gating, respiratory gating, or inversion times for DE which further increases operator/patient dependency. In addition, there is an increased post-processing burden due to the additional data generated in a 3D exam compared to conventional 2D CMR. There has been a lack of evaluation in large patient cohorts, and none of these methods have been demonstrated outside of the academic hospital setting. No end-to-end solution has yet been proposed which automates both image acquisition and post-processing, combining 3D isotropic cine and DE to effectively remove the need for a highly trained technologist while providing the key features of a CMR exam: anatomy, function, and tissue characterization.

We propose a fully automated CMR exam (AutoCMR) which offers a push-button, free breathing, whole thoracic exam with 3D isotropic cine and DE imaging and integrated analysis in less than 30 minutes. AutoCMR leverages multidimensional physics and state-of-the-art AI-based algorithms to enable a complete end-to-end automated solution inclusive of image acquisition, reconstruction, and post processing that provides a summary of volume and function metrics. Notably, one key novel technical feature is an AI segmentation-based self-navigation algorithm capable of automatically identifying and resolving cardiac and respiratory motion, which facilitates robust free breathing and multidimensional acquisition. With its simplified protocol, AutoCMR can be implemented by MRI technologists without CMR training. The inclusion and validation of automated whole-heart metrics addresses the need to reduce analytic burden implicit to 3D imaging. AutoCMR was rigorously validated in preclinical, academic, and community health center (CHC) settings against both same-session standard CMR and prior standard-of-practice clinical CMR studies. We finally briefly explore how AutoCMR has the potential to enable other cardiovascular personalized medicine technologies using AutoCMR’s underlying isotropic functional whole-heart multidimensional imaging data.

## Results

### AutoCMR Protocol, Reconstruction, And Biomarker Extraction

**Figure 1:**
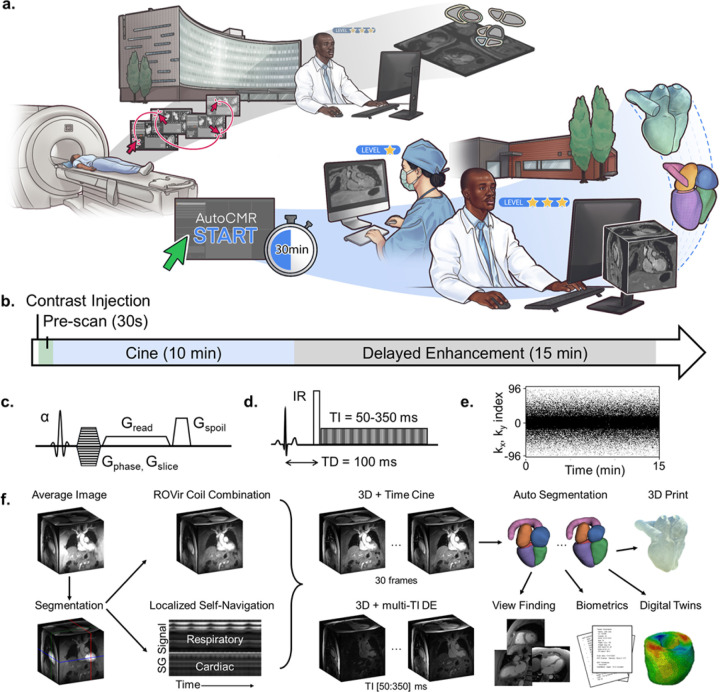
AutoCMR provides 4D isotropic cardiac MRI with automated analysis in a push-button exam **a,** Conventional CMR (top) relies on complicated imaging protocols with hundreds of decision points, yielding several sets of non-isotropic 2D images at fixed slice locations that provide only partial coverage of the heart. With its complex image acquisition and interpretation, CMR is currently limited to Academic Hospitals with access to expert CMR personnel. AutoCMR (bottom) is a push-button CMR exam, providing 4D morphology, function, and tissue characterization with isotropic whole heart coverage and automated analysis. With its simplified image acquisition and interpretation, AutoCMR enables advanced cardiac imaging in community health centers and outpatient imaging centers. **b,** The 30-minute AutoCMR exam begins with contrast injection and a 30s pre-scan, followed by a 10-minute 3D isotropic Cine acquisition and a 15-minute 3D isotropic Delayed Enhancement (DE) acquisition. **c,** All sequences in the AutoCMR exam employ a 3D spoiled gradient echo readout with an axial readout and a flip angle α < 15°. **d,** The DE acquisition is pulse-oximeter triggered with a 100 ms trigger delay (TD), followed by a non-selective inversion pulse and 400 ms of data acquisition, enabling image reconstruction with multiple inversion times (TI). **e,** k-space coverage is achieved with Cartesian Gaussian-random sampling in phase-encoding and slice select directions (k_x_, k_y_). **f,** (left to right) Image reconstruction utilizes all acquired cine data to generate an average image, which is automatically segmented to localize the heart. The heart’s position is used to perform targeted region-optimized virtual coil combination (ROVir)^28^ to reduced aliasing and self-navigation with self-gating (SG) signals in the region of the heart. 3D Cine data is respiratory corrected and time-resolved into 30 cardiac frames. 3D DE data is respiratory corrected and reconstructed with inversion times from 50–350 ms. All 30 cardiac frames of the cine data are automatically segmented, and segmentations are used to automatically generate traditional 2D cardiac views and biometric summaries. Segmentations further facilitate 3D printing and the generation of digital twins.

The AutoCMR exam begins with a less than 1 minute pre-scan during the injection of a gadolinium-based contrast agent, followed by 10 minutes of free-running 4D isotropic cine imaging and 15 minutes of pulse-oximeter triggered 3D isotropic delayed enhancement (DE) imaging (Fig. 1b–d). Acquisitions were designed and implemented using the open-source software Pulseq^29,30^. All data is collected free breathing, with total scan time of 30 minutes. With its streamlined design, the AutoCMR protocol has significantly fewer required technologist interactions and less variable scan duration compared to conventional CMR (Extended Data Figure S1). In 125 patients studied, 30% had clinical CMR exams where localization, 2D cine, and DE required longer than 35 minutes, while the longest AutoCMR exam was 32 minutes.

Prior to image reconstruction, the acquired AutoCMR data must be sorted according to motion and contrast states. Cardiac localized self-gating and principal component analysis are used to bin cine data into 30 cardiac frames and a single respiratory motion state (Fig. 1f)^31^. Extended Data Figure S2 shows high correlation of measured heart beats from AutoCMR self-gating compared to standard ECG gating, as well as qualitatively similar reconstructed motion patterns of AutoCMR in a single slice image compared to standard ECG triggered cine. DE data are similarly respiratory motion binned^31^ and divided into 80ms windows with inversion times (TI) ranging from 80 to 340 ms to provide multiple inversion-recovery contrasts. Images are then reconstructed with region-optimized virtual coils (ROVir)^28^, compressed sensing^32,33^, and low rank denoising^34^. Phantom studies demonstrate that AutoCMR can resolve ≤ 1.6mm^3^ isotropic features and provides the expected variation in contrast for DE images at varying TI (Extended Data Figure S3). Sample videos showing AutoCMR images in 4D are provided in Extended Data Video S4.

Cine images across all cardiac frames are auto-segmented for LV myocardium, LV blood pool, RV, left atrium (LA), right atrium (RA), and aorta using a Swin-UNETR^35^. The human segmentation model, trained on 83 datasets from 47 patients in a mixture of left ventricular end diastole (LVED) and end systole (LVES) cardiac motion states and validated on 10 datasets from 5 patients, achieves Dice-Sorensen scores > 0.8 for all segments when compared to a test set of 20 manually segmented images from 10 patients (Extended Data Figure S5). A separate swine segmentation model, fine-tuned from the human model using 8 training datasets from 4 pigs, also achieves Dice-Sorensen scores > 0.8 for all segments in a validation set of 2 manually segmented images from 1 pig.

A natural extension for AutoCMR’s whole heart segmentation is the generation of an automated structured clinical phenotype summary of key cardiac imaging biomarkers, including end diastolic volume (EDV), end systolic volume (ESV), and ejection fraction (EF) of all four cardiac chambers, left and right ventricular cardiac output (CO), and left ventricular mass (LVM). In seconds after completion of segmentation, a tabulated summary of key cardiac imaging biomarkers is automatically generated based on standard landmark rules (see Supporting Information for example biomarker summary). Segmentations are additionally also used to generate relevant landmarks to prescribe standard 4 chamber (4CH), 2 chamber (2CH), and short axis (SAX) views (Extended Data Figure S5m–p).

### Validation in a Preclinical Animal Model

In a conventional CMR exam, thick-slice (8mm) images are acquired interspersed with gaps to provide coverage over the extent of the LV while limiting the required number of patient breath-holds and the overall scan duration. The resulting piecemeal and LV-focused coverage of the heart provided by 2D CMR in patients is inadequate to validate 3D isotropic whole-heart imaging like that provided by AutoCMR. To overcome these limitations, AutoCMR was validated in a pre-clinical model, with thin-slice, gap-free, whole-heart 2D cine for functional assessment and histological validation of DE scar quantification. We scanned 10 swine with induced myocardial infarction (MI) on a 3T MR system under Institutional Animal Care and Use Committee approval. AutoCMR 3D isotropic cine and DE were compared with thin-slice (2mm) conventional 2D cine covering the whole heart and conventional 2D DE obtained in the same scanning session. We further validated scar size with 2,3,5-triphenyltetrazolium chloride (TTC) staining in histological slices of the LV. Cine data from 5 of these studies were used to train and validate a swine segmentation model. The model was then applied to the remaining 5 cine datasets to extract imaging biomarkers for comparison to 2D CMR. Scar quantification was performed in all 10 subjects.

**Figure 2:**
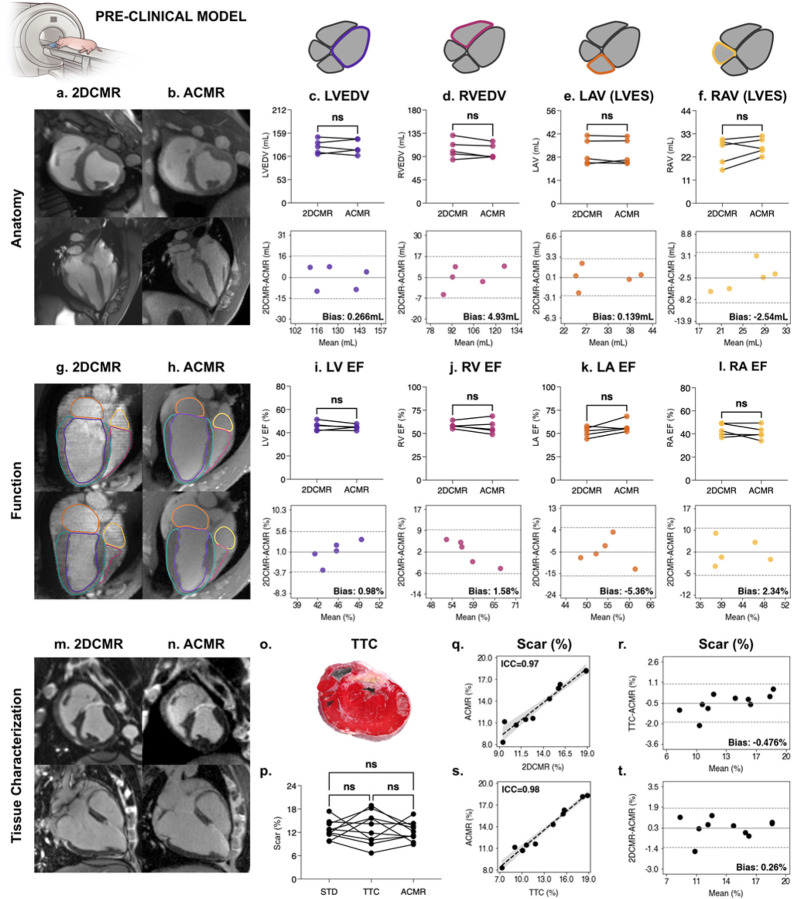
AutoCMR imaging biomarkers are comparable to 2DCMR in a pre-clinical model with 10 MI-swine. **a,** Cine images in short-axis (top) and 4-chamber (bottom) views acquired with conventional 2D CMR (2DCMR). **b,** AutoCMR (ACMR) cine images reformatted into short-axis (top) and 4-chamber (bottom) views, with visible antero-septal wall enhancement corresponding to infarcted tissue. **c-f,**Volumes of all four chambers of the heart acquired from 2DCMR and ACMR cine demonstrate no significant difference (p>0.05) using paired t-tests (top) and low bias in Bland-Altman analysis. **g,** Manually segmented 2DCMR cine images acquired in the short-axis plane with 2mm slice thickness and reformatted into the 4-chamber view to show LV, RV, LA, RA, and myocardial segmentations in diastole (top) and systole (bottom). Automatically segmented ACMR cine images in diastole (top) and systole (bottom) and reformatted into 4-chamber views, showing comparable LV, RV, LA, RA, and myocardial segmentations to 2DCMR. **i-l,** Functional analysis in all four chambers of the heart using ACMR cine demonstrates no significant difference in EF compared to 2DCMR (p>0.05) using paired t-tests (top). In Bland-Altman analysis (bottom), left and right ventricular and atrial EF have <2% and <6% bias, respectively. **m-n,**Delayed enhancement imaging with conventional 2DCMR (**m**) and ACMR (**n**) in short-axis (top) and long-axis (bottom) views demonstrate transmural septal wall enhancement. **o,** TTC staining showing septal wall scaring in agreement with DE imaging. **p-t,** Quantification of the percent scar using manual segmentation of TTC staining, 2DCMR DE and ACMR DE images shows no significant difference (p>0.05) between the techniques using One-Way ANOVA and Dunnett’s Multiple comparisons (**p**), high correlation (ICC≥0.97) of ACMR data with TTC staining (**q**) and 2DCMR (**s**), and bias <1% for ACMR compared to TTC staining (**r**) and 2DCMR (**t**) in Bland-Altman analysis. LAV: LA volume in LVES. RAV: RA volume in LVES.

AutoCMR cine achieved isotropic through-plane resolution and temporal feature fidelity when compared to thin-slice 2D cine imaging in the porcine model (Fig. 2a–l, Extended Data Figure S6a–g). In two-sided paired t-tests, AutoCMR and thin-slice 2D cine showed no significant difference (p > 0.5) in any anatomic biomarkers, including LVEDV, RVEDV, LA volume, RA volume, and LV mass (LVM). LVEDV and LA volume achieved less than 1 mL in bias, while RVEDV and RA volume measured slightly more at 4.93 mL and −2.54 mL, respectively. LVM bias was similarly low at 2.2 g. Functional measures, including ejection fraction (EF) and cardiac output (CO), likewise showed no significant difference (p > 0.05) compared to 2D CMR across all four cardiac chambers in two-sided paired t-tests. There was minimum bias in EF in all 4 cardiac chambers, with all LV EF values within 2–3%, and similar agreement in CO.

AutoCMR DE was comparable to conventional 2D DE imaging and TTC staining in both qualitative enhancement area and scar quantification (Fig. 2m–t). One-way ANOVA with Dunnett’s multiple comparisons tests revealed no significant difference in scar quantification between the three techniques. In direct comparisons, AutoCMR scar quantification was highly correlated (ICC = 0.98, 0.97) and minimally biased (< 1%) compared to conventional 2D DE and TTC.

### Evaluation in Patients at an Academic Hospital

For evaluation in a large patient cohort, AutoCMR was performed at an academic hospital where trained CMR technologists were available to perform same day 2D CMR for the correlation of anatomical and functional measures. Although the contrast-based time dependence of DE imaging prevented same day acquisition of AutoCMR and 2D DE, the unlikelihood of significant scar morphology or extent changes over a limited time enabled comparison to previously acquired clinical 2D DE.

We scanned 179 patients who previously received a contrast CMR study for any reason under a Cleveland Clinic Institutional Review Board approved protocol on a 3T MR system operated by CMR technologists with over 20 years of training. Standard localizers and 2D breath-held cine were performed, followed by AutoCMR in a single scanning session. 27 subjects were excluded in final analysis due to failed automated self-gating (12), patient positioning with the heart not fully covered by the FOV (6), acquisition error (4), segmentation model failure (4), and gating failure of conventional 2D CMR (1). The final cohort of 152 patients included 29 subjects with known hypertrophic cardiomyopathy (HCM), 12 subjects with diagnosed ischemic disease, and 52 patients with no known cardiovascular disease (Extended Data Table S7).

#### Volume and Function Evaluation

Excluding the 52 patients in the auto-segmentation training and validation datasets, four-chamber metrics extracted from automated AutoCMR cine segmentations and manually-corrected automated segmentations of same-day 2D CMR cine images for the remaining 100 patients were compared using intra-class correlation and Bland-Altman analysis.

**Figure 3:**
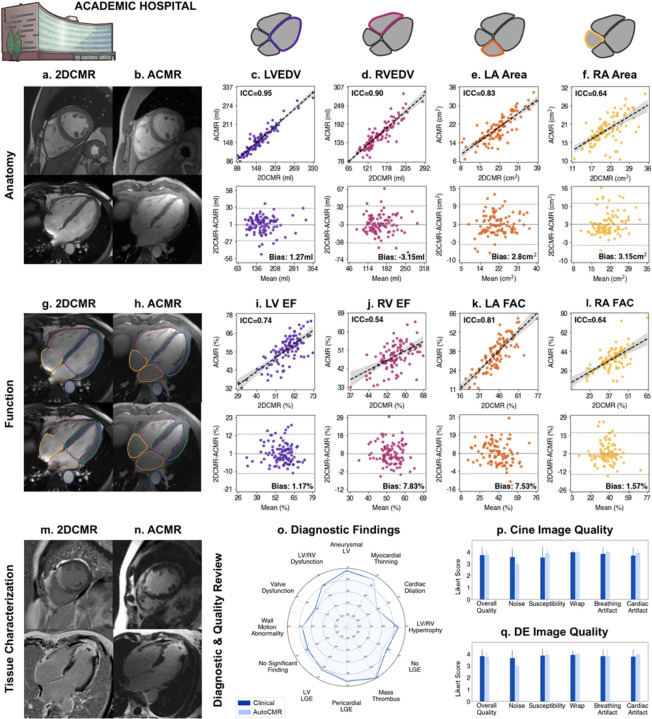
AutoCMR imaging biomarkers are comparable to 2DCMR in 100 patients scanned at an academic hospital. **a-b,** Cine images in short-axis and 4-chamber views acquired with conventional 2DCMR (**a**) and AutoCMR (**b**) in the same scanning session. **c-d,** LV and RV volumes in end diastole calculated from 3D isotropic AutoCMR cine and 2DCMR short-axis cine with 8-mm slice thickness and 2mm gap are highly correlated (top) with ICC≥0.90 and have bias ≤4mL in Bland Altman analysis (bottom). **e-f,** Atrial areas are compared using 4-chamber 2DCMR cine and AutoCMR cine reformatted into 4-chamber views. LA and RA areas demonstrate good (ICC=0.83) and moderate (ICC=0.64) correlation (top), respectively, and low bias in Bland-Altman analysis (bottom). **g-h,** 2DCMR and AutoCMR cine images in diastole (top) and systole (bottom) are shown in 4-chamber views with LV, RV, LA, RA, myocardium, and aorta segmentations. 2DCMR segmentations were semi-automatic, with corrections by a trained cardiologist, while AutoCMR segmentations were performed automatically without any user corrections. **i-j,**3D AutoCMR and 2DCMR LV and RV ejection fraction demonstrate moderate correlation (ICC=0.74, 0.54). Bland-Altman analysis shows low bias in LVEF (1.17%) and moderately low bias in RVEF (7.83%). **k-l,** LA and RA function is quantified using fractional area change (FAC) calculated from 4-chamber imaging views, which shows good (ICC=0.81) and moderate (ICC=0.64) correlation, respectively. In Bland-Altman analysis, LA and RA FAC are observed to have moderately low to low bias (7.53%, 1.53%). **m-n,** Delayed enhancement imaging in short-axis and 4-chamber views acquired with 2DCMR and AutoCMR, showing transmural delayed enhancement in the septum and apex and an apical thrombus. **o,**Significant cine and DE findings in a review of 30 patients, showing comparable accuracy between 2DCMR (dark blue) and AutoCMR (light blue) compared to ground truth in all categories. **p-q,** Image quality review of 30 patients demonstrating comparable 2DCMR and AutoCMR cine and DE image quality with minimal artifacts.

AutoCMR provided high resolution, whole-thoracic anatomic imaging comparable with standard CMR (Fig. 3a–f, Extended Data Figure S6h–p). Measurements of end diastolic and end systolic ventricular volumes were highly correlated with ICC of 0.95, 0.94 for LV and 0.90, 0.88 for RV, respectively, with a slight negative bias in RVESV (−13.9mL) in Bland-Altman analysis. Atrial areas calculated using 4-chamber views yielded moderate to moderate high ICC with 0.83 ICC and 0.64 ICC for LA and RA, respectively. Although highly correlated (ICC = 0.94), AutoCMR images have limited myocardium-fat delineation, contributing to LVM overestimation (Extended Data Figure S6i). Measurement of aortic diameter was also highly correlated in a subset of patients with prior clinical CT/MR angiography studies (Extended Data Figure S6u).

Analysis of heart function revealed consistent results between AutoCMR and conventional 2D cine (Fig. 3g–l, Extended Data Figure S6q–t). AutoCMR LVEF, RVEF, LA fractional area change (FAC), and RA FAC have moderate ICC of 0.74, 0.54, 0.81, and 0.64, respectively. LVEF and RA FAC also show minimum bias with both being less than 2%. Reduced correlation of AutoCMR and 2D CMR for RVEF with bias of 7.68% reflects the complex anatomy of the RV and the resulting dependence of the calculated 2D CMR RV volume on SAX slice positioning and orientation. To eliminate inherent discrepancies in comparing 2D to 3D data, we further evaluated the captured LV motion by manually measuring change in ventricular area in a single matched mid-ventricular slice, yielding highly correlated (ICC = 0.95) LV FAC with bias less than 2% (Extended Data Figure S2c–f).

#### Diagnostic and Image Quality Review

AutoCMR tissue characterization, diagnostic potential, and image quality was evaluated in comparison to previously acquired clinical 2D cine and DE images in a subset of 30 patients (Extended Data Table S7) by two, level 3 CMR certified readers.

In the diagnostic review of significant cine and DE findings (Fig. 3o, Supplemental Information), AutoCMR and clinical 2D images yielded comparable accuracy compared to the ground truth. Without volumetric and functional measures and with limited ability to manipulate the provided images during the review, assessment of valve dysfunction yielded moderate accuracy for both image types (0.60, 0.62). Ventricular dilation and dysfunction similarly yielded good agreement between AutoCMR and clinical 2D images, but some discrepancy compared to the ground truth (Cardiac Dilation: 0.60, 0.60; LV/RV Dysfunction: 0.72, 0.82). Identification of regions of late gadolinium enhancement using AutoCMR DE and clinical 2D DE images again demonstrated comparable accuracy compared to the ground truth, achieving accuracy of 0.80, 0.83 in identifying LV LGE positive cases and 0.72, 0.78 in identifying LGE negative cases, respectively. McNemar’s test comparing accuracy based on AutoCMR and clinical 2D CMR images against the ground truth revealed no significant difference in any categories (p > 0.05).

The readers rated image quality of AutoCMR cine (Fig. 3p) and DE (Fig. 3q) studies to be comparable to conventional 2D CMR (Likert score of 3.74 to 3.72 for cine and 3.73 to 3.80 for DE), with AutoCMR having slightly higher incidence of noise (Likert score of 2.93 to 3.57 for cine and 2.97 to 3.65 for DE), but reduced susceptibility, breathing, and cardiac motion artifacts. All AutoCMR images were free of diagnostically compromising artifacts, with no images rated as non-diagnostic.

### Implementation and Feasibility at a CHC with no prior CMR experience

Because lack of CMR-trained technologists is a known barrier to growth^6^, we aimed to demonstrate the feasibility of implementing AutoCMR in a Community Health Center (CHC) with no prior CMR experience. 35 patients who previously received a clinical CMR exam were recruited and scanned using AutoCMR on a 1.5T MR system at a local CHC by technologists with no CMR training. Initial testing and sequence development were performed in 5 patients, who were excluded from final analysis. An additional 5 subjects were excluded due to invalid placement of the body coil resulting in an incomplete FOV (1), failed automated self-gating (1), segmentation model failure (2), and poor ECG gating of clinical 2D CMR (1). The final cohort of 25 patients included 6 subjects with known hypertrophic cardiomyopathy (HCM), 3 subjects with diagnosed ischemic disease, and 10 patients with no known cardiovascular disease (Extended Data Table S7).

#### Volume and Function Evaluation

**Figure 4:**
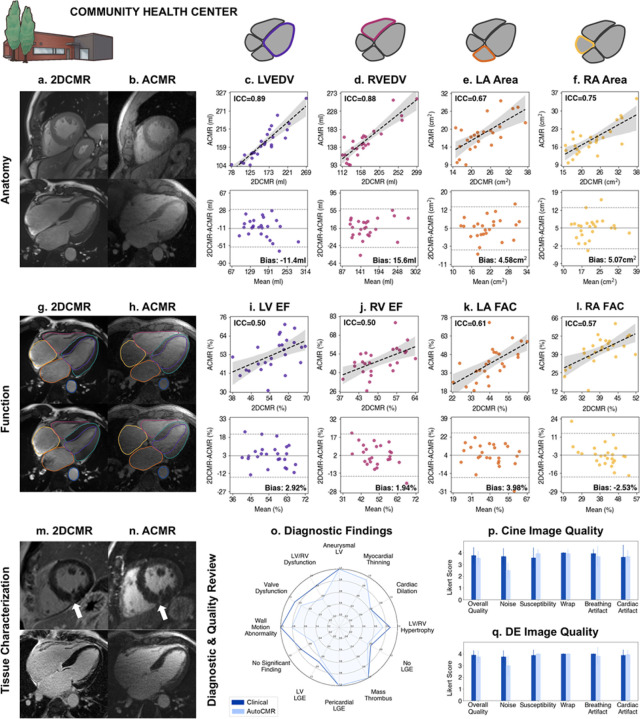
AutoCMR enables CMR in a Community Health Center with no CMR training. **a, b,** 2DCMR cine acquired at an Academic Hospital on a 3T MRI by trained CMR technologists is compared to AutoCMR cine acquired at a CHC with a 1.5T MRI by technologists with no CMR training in short-axis (top) and 4-chamber (bottom) views. **c, d,** Left and right ventricular volumes in end diastole demonstrate good correlation (ICC=0.89, 0.88) and moderately low bias (−11.4, 15.6 mL) in Bland-Altman analysis, which can be partially attributed to the time between scanning sessions. **e, f,** LA and RA area calculated using 4-chamber views demonstrate moderate reliability (ICC=0.67, 0.75) and similarly moderate bias (4.58, 5.07 cm^2^) in Bland-Altman analysis. **g, h,** Segmented cine images in diastole (top) and systole (bottom) are shown in 4-chamber views with LV, RV, LA, RA, myocardium, and aorta segmentations. 2DCMR images were segmented semi-automatically with corrections by trained cardiologists. AutoCMR images were automatically segmented, and results are presented without manual modifications. **i-l,** Functional metrics in all four chambers (**i,** LV EF, **j,** RV EF, **k,** LA FAC, **l,** RA FAC) calculated using AutoCMR performed at the CHC and clinical CMR performed at an academic hospital demonstrate moderate correlation (ICC=0.50, 0.50, 0.61, 0.57) and less than 5% bias (2.92%, 1.94%, 3.98%, −2.53%). **m, n,** Delayed enhancement imaging in short-axis (top) and 4-chamber (bottom) views reveals minimal focal enhancement at the inferior right ventricular insertion point (white arrows) in both 2DCMR and AutoCMR. **o-q,** Significant diagnostic findings (**o**) in a review of 8 patients, showing largely overlapping accuracy between 2DCMR acquired at an academic hospital (dark blue) and AutoCMR acquired at a CHC (light blue). **p-q,** Image quality review of an 8-patient subset demonstrating minimal observed artifacts and comparable although decreased cine and DE image quality for CHC AutoCMR compared to 2DCMR.

All CHC AutoCMR comparisons are made relative to clinical 2D CMR previously acquired at an Academic Hospital during standard of care as no CMR technologist was available to do a same day comparative study. AutoCMR cine imaging and auto-segmentation yielded high ICC of anatomic volumes in all 4 chambers compared to the previously acquired clinical exam (LVEDV = 0.89, RVEDV = 0.88, LA Area = 0.67, RA Area = 0.75) (Fig. 4a–f, Extended Data Figure S6v–ad). Functional metrics were less correlated compared to results in an Academic Hospital as expected due to the elapsed time between clinical and AutoCMR exams in the CHC study, but still achieved moderate ICC (LVEF = 0.50, RVEF = 0.50, LA FAC = 0.61, RA FAC = 0.57) and EF bias less than 4% (Fig. 4g–l, Extended Data Figure S6ae–ah).

#### Diagnostic and Image Quality Review

In a subset of 8 patients (Extended Data Table S7), two level 3 CMR readers evaluated AutoCMR and previously acquired clinical 2D cine and DE images for cardiac function and significant cine findings (Fig. 4o), late gadolinium enhancement and significant DE findings (Fig. 4o), and image quality (Fig. 4p–q). In this small cohort feasibility study, significant cine findings identified using AutoCMR images were generally less accurate compared to the ground truth than those identified with clinical 2D CMR images (see Supplemental Information). Accuracy in identifying late gadolinium enhancement was comparable for AutoCMR and clinical 2D images, with (0.63, 0.69) accuracy in identifying LV LGE positive cases and (0.56, 0.63) accuracy in identifying LGE negative cases, respectively.

The image quality of AutoCMR cine at the CHC was rated generally worse compared to AutoCMR images acquired in the academic setting (Likert score of 3.55 to 3.74), while DE images were similarly rated in the CHC and 3T academic settings (3.75 to 3.73). AutoCMR images acquired at the CHC with a 1.5T system were nosier than the clinical images (2.50 to 3.69) and contained some breathing artifacts (3.69 to 3.94). However, with its GRE readout AutoCMR is robust to banding artifacts (3.94 to 3.56).

### AutoCMR for next generation patient analytics as proof-of-concept

**Figure 5:**
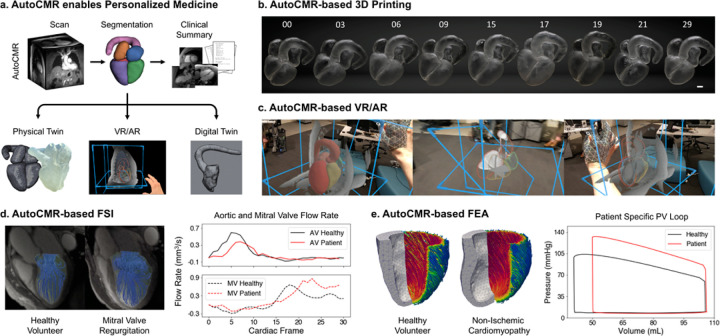
AutoCMR enables personalized medicine and digital twins. **a,** AutoCMR provides 3D isotropic cine images with high spatial (1.6mm) and temporal (30 frames per heart beat) resolution. Automated segmentation of all 30 cardiac frames enables automated view finding of traditional 2DCMR views and volumetric and functional reporting. Segmentations can readily be used to generate physical twins through 3D printing, digital twins using FSI and FEA modeling, and VR/AR visualizations (e.g. HoloLens). **b,** 3D prints of 9 of the 30 cardiac frames (scale bar at 10 mm) demonstrate the scalable and flexible nature of printing with AutoCMR images and automated segmentations. **c,**Images from a VR/AR video demonstrating how AutoCMR data can be visualized into a mixed reality space with various levels of opaqueness (left and right) and slice planes (middle). **d,** FSI can be implemented directly on the 3D isotropic, 30-frame cardiac data provided by AutoCMR, without need for spatial interpolation as is often required with single frame CT data. Simulations show expected increase in mitral flow rate and decrease in aortic flow rate in the mitral valve regurgitation patient compared to the healthy volunteer. **e,** Similarly, FEA utilizes 30 cardiac frame data as input to simulations of LV pressure over time. Resulting pressure-volume (PV) loops show increased systolic pressure in the patient with LV hypertrophy and hypertension compared to the healthy volunteer.

As a proof-of-concept, AutoCMR’s time resolved 3D whole heart segmented data was utilized as input to three key personalized medicine technologies: 3D printed physical twins, virtual / augmented reality, and digital twins that rely on subject-specific geometric and temporal boundary conditions of the heart (Fig. 5a). To showcase an AutoCMR-enabled 3D printed physical twin, a cine segmentation in end diastole from an aortic valve regurgitation patient, including all four chambers of the heart and the aorta, was directly converted into an STL mesh using an open-source medical imaging slicing tool (3D Slicer)^36,37^. Support structures were determined using the commercially available automated software PreForm (Formlabs). All pre-processing was completed in 45 minutes, and the 3D print took 27 hours for the selected cardiac frame at full scale. Eight more cardiac phases across the cardiac cycle were 3D printed in the same automated manner to illustrate the flexibility in printing any of the 30 frames resolved by AutoCMR (Fig. 5b). As an alternative to 3D printing, AutoCMR cine images and automated whole-heart segmentations, saved as nifti and obj files respectively, were also used for direct visualization in an extended reality (XR) environment (CWRUXR, Cleveland, OH, USA) (Fig. 5c, Extended Data Video S8). Using tools provided by CWRUXR, it took less than 30 mins to prepare the first dataset for XR visualization and, with the established pipeline, new datasets can be added in minutes.

The 30-frame time resolved 3D segmented datasets for two patients (non-ischemic cardiomyopathy with moderate mitral valve regurgitation and suspected hypertrophic cardiomyopathy) and one healthy volunteer were converted into STL meshes and directly input into two established digital twin technologies: a commercially-available fluid structure interaction (FSI) model (Abaqus, Dassault Systèmes) (Fig. 5d) and a finite element analysis (FEA) model implemented in Abaqus (Dassault Systèmes) with a user-defined VUMAT material subroutine, incorporating boundary conditions derived from a subject-specific lumped-parameter circulation model calibrated in Python(Fig. 5e). For the FSI model using time resolved LV volume and mass segmentations, the computer simulated flow patterns show turbulence in the form of a classic mushroom flow pattern indicative of regurgitation compared with the healthy volunteer. Computer simulated flow over time plots reveal a severe reduction in the flow gradient across the mitral valve in the mitral valve regurgitation patient (MV peak flow: 0.87 mm^3^/s) compared with healthy volunteer (MV peak flow: 0.66 mm^3^/s). For the FEA model using only the time resolved LV mass segmentation, the computer simulated pressure over time curves for the non-ischemic cardiomyopathy patient revealed a substantial increase in peak LV systolic pressure (133 mmHg) when compared with the healthy subject (104 mmHg), which is consistent with the patient’s history of hypertension, LV hypertrophy pathology^38^, and clinical blood cuff measurements (135 mmHg). With its high temporal resolution, AutoCMR is also shown to enable accelerated FEA simulation through fast determination of the unloaded geometry of the heart (Extended Data Figure S9).

## Discussion

AutoCMR is a 30-minute end-to-end automated CMR exam that delivers 4D morphological, functional, and tissue characterization of the whole heart in a single click without breath-holds. AutoCMR was rigorously validated in three cohorts including preclinical large animals, patients scanned at an academic hospital with over 40 years of CMR experience, and patients scanned at a community health center with no prior CMR experience. While providing a simplified CMR acquisition and automated analysis, we demonstrated that AutoCMR was comparable to conventional CMR in imaging biomarkers and human interpretation. With AutoCMR’s 3D whole thoracic coverage and temporal resolution of 30 frames per heartbeat, we further showcased that AutoCMR can enable next generation patient analytics including personalized digital twins, 3D printing, virtual reality, and automated structured biometric summaries. Taken together, AutoCMR greatly simplifies the cardiac MRI exam and its analysis promoting democratization of advanced cardiovascular imaging access.

MRI in the cardiothoracic region is particularly challenging due to respiratory and cardiac motion that necessitate gating and fast-imaging techniques, the abundance of tissue interfaces and prevalence of metal implants causing susceptibility artifacts, and highly variable anatomy and body habitus, requiring careful image planning to acquire desired cardiac views without wrap artifacts. For tissue characterization, the optimal inversion time for scar visualization depends on individual contrast dynamics and scan timing. As a result, technologists with specialized CMR training and the ability to respond to these challenges in real time are vital in obtaining diagnostic images^18^. To ensure ease of implementation in centers with no CMR expertise, AutoCMR was designed to ameliorate these challenges. With its spoiled gradient echo-based implementation, AutoCMR is intrinsically less susceptible to banding, flow, and metal artifacts compared to traditional bSSFP-based CMR (Extended Data Figure S10a). Wrap artifacts due to body habitus are handled using ROVir^28^ to suppress information from outside of the field of view (Extended Data Figure S10b). Arrythmia is observed and automatically addressed with self-gating, using adaptive binning according to each peak-to-peak interval and rejection of data acquired during intervals that are more than two standard deviations outside the mean duration (Extended Data Figure S10c). By offering a range of available inversion times, DE images can be retrospectively selected with the optimal contrast for observing enhancement, including diffuse LGE (Extended Data Figure S10d).

With the challenges of cardiothoracic imaging, CMR scan duration is long and variable (60–90 minutes), and the diagnostic quality of conventional CMR images is heavily dependent on expertly trained technologists. The Society for Cardiovascular Magnetic Resonance has recognized that the long scan times^17^ and the limited availability of well-trained CMR technologists^18^ stifle widespread operational use, particularly in community settings where MRI hardware is shared with non-cardiac imaging. There have been significant efforts to minimize CMR exam time by optimizing protocol^17^, minimizing cardiac coverage^39^, and automating view finding and prescription^22^. However, these efforts are still constrained by the inherent 2D breath-hold nature of conventional CMR and still requires a tech with high skill level in case automated scanning fails. AutoCMR inherently addresses all these problems by completely capturing the thoracic cavity in a 30-minute free-breathing exam, removing the need for technically difficult prospective slice positioning and laborious breath-holds and thereby removing the need for a highly trained technologist. The consistency of the AutoCMR scan protocol and duration enables tighter scheduling windows and increased number of slots, enabling scheduling on anatomy agnostic scanners.

AutoCMR offers more than just reduced logistic barriers to comprehensive cardiac imaging. Its underlying multidimensional data can be used to enable additional personalized medicine technologies, which rely on input data that faithfully captures the structure and function of a subject’s heart. Computational, virtual, and physical models of the heart have been extensively studied and proposed for simulated disease prediction, digital clinical trial, and surgical planning^40–42^. Currently, these models require significant interpolation of the input data either between slices (echocardiography or conventional CMR) or throughout the cardiac cycle (cardiac computed tomography) due to the intrinsic limitations of the underlying imaging technology. The interpolated image data requires segmentation for mesh generation, which is laborious if done manually and ultimately limits scalability and clinical delivery of the technology. We feasibly showcased such potentially intractable pre-processing is not necessary when using AutoCMR as input to these personalized medicine technologies as it offers true isotropic and time-resolved functional imaging and segmentation of the heart. AutoCMR gives an opportunity for these technologies to have a natural stream of input data directly from existing clinical infrastructure allowing them to be more easily operationalized in a clinical setting and promoting a potentially scalable method.

This work presents the feasibility of a nearly automated pipeline for an abbreviated volumetric, free breathing CMR exam suitable for democratized cardiovascular imaging and thus contains several limitations. Our initial diagnostic human interpretation review had several limitations, including restricted functionality of the image viewer (i.e. lack of control over image contrast, brightness, and zoom), no inclusion of medical history or volume and function metrics, and the use of single reader clinical reports as ground truth. Despite these limitations, the two blinded reviewers demonstrated AutoCMR to be consistent with conventional CMR in both the major academic hospital and community health settings. More extensive clinical validation of AutoCMR is necessary for eventual clinical translation, potentially in the form of a randomized control trial utilizing real-world reading conditions including commercially available image viewer software and ground truth from a review panel. In addition, AutoCMR in its current form provides only the core CMR sequences of cine and DE. Other important CMR sequences such as perfusion, relaxometry (e.g. T1 and T2 mapping), and flow are features important to the clinical diagnosis and prognosis of advanced indications such as heart failure and valvular disease. Although not in this initial version, it is possible to incorporate such sequences in future iterations via automated view finding with slice prescription^22^ or integration of other promising multidimensional techniques^43^. Lastly, the computational resources needed to perform the automated end-to-end image reconstruction and segmentation currently deters real-time monitoring throughout the exam. Currently, imaging technologists require immediate feedback on image quality to review slice positioning, cardiac gating, breathing artifacts, and other artifacts which may impact diagnostic quality. We demonstrated that these artifacts are less of a concern with AutoCMR due to the gradient-echo acquisition and retrospective reconstruction. Although approximately 7% of our studies were removed from analysis due to respiratory or cardiac motion artifacts using the fully automated pipeline, all cases could pragmatically benefit from manual modification of the self-navigated motion binning. However, issues such as initial mispositioning of the receiver coils or large bulk motion shifts may still corrupt the AutoCMR study. In the future, highly efficient deep learning-based reconstruction may be used for inline real-time reconstruction with lower requirements for edge computational resources.

Our results represent a vital step towards the simplification of advanced cardiac imaging examinations for automated evaluation of cardiac anatomy, function, and tissue characterization. By lowering intractable technical and institutional barriers, AutoCMR has the potential to enable routine cardiac MRI examinations in hospital settings beyond major academic institutions. AutoCMR’s underlying data consists of rich multidimensional information, which can additionally act as a scaffold for advancing current personalized medicine methods that rely on subject-specific spatial, temporal, and disease features as input.

## Methods

### Sequence Design

The AutoCMR acquisition was developed using Pulseq^29,30^, an open-source and hardware-independent framework that supports implementation on scanner platforms from multiple vendors. The 30-minute exam (Figure 1b) begins with a slow infusion^44^ gadolinium-based contrast injection at a rate of 0.25 mL/s. A large field of view (FOV=500mm), low-resolution (5mm isotropic) pre-scan image is acquired in 30s with a spoiled gradient echo acquisition. 3D isotropic cine data with 1.6mm resolution and a 307mm FOV is continuously acquired for 10 minutes using a spoiled gradient echo acquisition (Figure 1c) (Academic hospital: TR/TE=4.0/2.9ms, FA=12°, G_max_=40 mT/m, slew_max_=150mT/m/ms; CHC: TR/TE=4.9/3.0ms, FA=12°, G_max_=20 mT/m, slew_max_=100mT/m/ms). Delayed enhancement imaging data is collected in approximately 15 minutes with a pulse-oximeter gated inversion recovery sequence and a spoiled gradient echo readout (Figure 1d) (resolution=1.6mm isotropic, FOV=307mm, scan duration=900 heartbeats, trigger delay=100ms, acquisition window=400ms; Academic Hospital: TR/TE=3.7/2.6ms, FA=10°, G_max_=40 mT/m, slew_max_=150mT/m/ms; CHC: TR/TE=4.7/2.9ms, FA=10°, G_max_=20 mT/m, slew_max_=100mT/m/ms). Slight modifications in sequence parameters for implementation at the Community Health Center (CHC) are the result of gradient hardware limitations. While reduced gradient capability results in increased TR, total scan time at the CHC and the Academic Hospital is fixed. All data is collected using Gaussian random sampling on the Cartesian grid (Figure 1e) with an axial readout. Self-gating^31,45^ lines through the center of k-space are acquired after every 10^th^ imaging line during cine acquisition and once per heartbeat in the DE acquisition.

### Image Reconstruction

An average initial image is calculated from all cine data and is automatically segmented to localize the heart (Figure 1f). Segmentations are used to define the area of interest for region-optimized virtual combination^28^, which reduces aliasing-related wrap from regions outside the field of view (Extended Data Figure S9b). Initial segmentations are also used to automatically select self-gating (SG) lines located near the LV for principal component analysis^31,45^. For cine data, frequency analysis automatically selects principal components corresponding to respiratory (< 0.6 Hz) and cardiac (> 0.6 Hz) signals. A Savitzky-Golay filter^46^ is applied to the selected cardiac signal prior to peak detection. Cine data is binned into a mode respiratory phase using respiratory signal amplitude and 30 cardiac frames using peak detection and rejection of data within RR-intervals that exceed a difference in duration of more than two standard-deviations compared to the mean. DE data is similarly binned into a mode respiratory phase and sorted by inversion time (TI) with 80 ms windows to generate images reconstructed with TI times ranging from 80ms to 340ms in steps of 10ms. 3D cine and DE images are reconstructed with slice-wise compressed sensing using SENSE^32,47–49^ and total variation^33^ in time, followed by multi-scale low rank^34^ denoising. DE images are initially generated as magnitude (MAG) images. DE images with phase-sensitive (PSIR) contrast are additionally generated by detecting the null-point per pixel using multi-TI data. Image reconstruction is performed in Python using Sigpy (Version 0.1.23) in less than one hour using a Nvidia A100 GPU (Nvidia, Santa Clara, California, USA).

### Post-Processing

Following image reconstruction, all 30 cardiac frames of the AutoCMR cine data are automatically segmented in less than 3 minutes using a Nvidia V100 GPU (Nvidia, Santa Clara, California, USA). Segmentations are used to calculate LV, RV, LA, RA, myocardial, and aortic volumes throughout the cardiac cycle. Calculated LV volumes are used to automatically determine LV end systole (LVES) and LV end diastole (LVED). Combined with the recorded heart rate determined during image reconstruction, segmented volumes are used to automatically calculate the following functional metrics: LV ejection fraction (EF), RV EF, LA EF, RA EF, LV cardiac output (CO), and RV CO. Myocardial volume was used to calculate left ventricular mass (LVM) in LVED. An automated volumetric and functional summary is generated by summarizing the calculated metrics and comparing them to established normal values in a standardized text file.

Segmentations are additionally used to determine cardiac landmarks including the center of mass and boundaries of each of the cardiac chambers, the apex of the heart, and the mitral, aortic, and tricuspid valve planes. From these landmarks, 2-chamber, 4-chamber, and short-axis views are automatically generated (Extended Data Figure S5m–p).

### Segmentation Model

#### Human segmentation model

Automated segmentation is performed with a Swin-UNETR model^35^ in 5s per 3D volume using a Nvidia V100 GPU (Nvidia, Santa Clara, California, USA). The segmentation model was trained on manually segmented AutoCMR cine data in left ventricular end diastole (LVED) and left ventricular end systole (LVES) from the human cohort scanned at an Academic Hospital. Manual segmentations of the left ventricle (LV), right ventricle (RV), left atrium (LA), right atrium (RA), myocardium, and aorta were performed in 3D Slicer^36,37^ and reviewed by a cardiologist. Manual segmentations from 46 unique patients (83 total frames), 5 unique patients (10 total frames), and 10 unique patients (20 total frames) were used for training, validation, and testing, respectively. The segmentation model was evaluated on the test set using Dice-Sorensen scores and Bland-Altman analysis of volume (Extended Data Figure S5a–l).

#### Swine segmentation model

The auto-segmentation model for the pre-clinical model was finetuned from the pre-existing weights of the human auto-segmentation model. During the finetuning process, the entire neural network was utilized to train on manually segmented LVED and LVES swine data, which consisted of 4 subjects (8 frames) in the training set, and 1 subject (2 frames) in the validation set. The resulting Dice-Sorensen score for the validation set is 0.859 ± 0.058 over 2 frames and 6 labels.

### Phantom Validation

Phantom experiments were performed on a 3T Cima.X (Siemens Healthineers) using an 18-channel body coil (Extended Data Figure S3).

A custom resolution phantom was designed in SolidWorks and printed on a FormLabs SLA 3B+ printer using a clear resin v4 at 50 μm layer resolution. It contains four 3×3 grids providing resolutions of 2.4mm, 2.0mm, 1.6mm, and 1.0mm. The resolution phantom was submerged in water mixed with one drop of Gadopiclenol (Elucerim^™^) and scanned using the complete AutoCMR protocol with a simulated heart rate of 60 beats per minute. Resolution phantom images were interpolated by a factor of 2 and line plots were generated through each of the resolution grids.

The 3T T1mes phantom^50^ was additionally scanned using the complete AutoCMR protocol with a simulated heart rate of 60 beats per minute. Voxels were manually chosen in each of the 9 regions of interest (T1=250, 294, 424, 451, 555, 1010, 1260, 1499, 1872 ms) and signal intensity from the DE PSIR images were plotted versus inversion time to show inversion recovery curves.

### Animal Model

All animal studies were completed according to approved protocols and standards of care set forth by the Cleveland Clinic Animal Care and Use Committee. The pre-clinical porcine model includes ten adult male Yorkshire swine. Myocardial infarct (MI) was induced with a percutaneous transluminal coronary angioplasty dilation catheter that was inflated for 120 minutes to occlude flow between the first and second diagonal branch of the left anterior descending coronary artery.

#### Imaging

Imaging was performed 4–6 weeks post-MI on a 3T Cima.X (Siemens Healthineers) using an 18-channel body coil. During imaging, anesthesia was maintained using 1–5% isoflurane and 2–20 mg/kg/hr propofol. AutoCMR pre-scan and cine imaging was acquired immediately following gadoterate meglumine (Dotarem^™^) or gadobutrol (Gadavist^™^) contrast injection at a dose of 0.2 mmol/kg. Conventional 2D DE imaging was performed with a free-breathing, motion corrected^51^ acquisition in 2-chamber, 3-chamber, 4-chamber, and short axis views (PSIR, TR/TE=4.3/1.7ms, FA=12°, 1.4mmx1.4mmx5.0mm, 12 averages), followed by AutoCMR DE. Conventional 2D cine imaging was acquired in the short-axis plane with 2mm slice thickness and slices covering the whole heart (spoiled gradient echo, TR/TE=6.1/3.4ms, FA=12°, 1.6mmx1.6mmx2mm, 4–6 averages).

#### TTC Staining

Animals were sacrificed immediately following the MRI exam. Harvested hearts were frozen for 2 hours, then cut into 5 SAX slices from apex to base. Slices were stained with 1% 2,3,5-triphenyltetrazolium chloride (TTC) solution at 37°C for 20 minutes, followed by 10% formalin fixation for 20 minutes. Pictures were taken of each stained slice. The LV and scar were manually segmented on the resulting images, and scar size was calculated as a percentage of total pixels using ImageJ^52^.

#### CMR Analysis

Manual segmentation of the LV, RV, LA, RA, myocardium, and aorta in AutoCMR (ACMR) and thin-slice, conventional 2D (2DCMR) cine data was performed using 3D Slicer^36,37^. 2DCMR data was manually segmented in LVED and LVES. Manual segmentations of LVED and LVES AutoCMR cine data from 5 pigs were used to train and validate the swine auto-segmentation model. The model was then applied to the 30-frame AutoCMR cine data for the remaining 5 subjects. The resulting manual 2DCMR and automated ACMR segmentations were used to calculate the following volumetric and functional parameters: LVED, LVES, LVEF, LVCO, LVM, RVED, RVES, RVEF, RVCO, LA Volume in LVED, LA Volume in LVES, LA EF, RA Volume in LVED, RA Volume in LVES, and RA EF, where LA and RA EF are calculated as (Volume in LVES – Volume in LVED) / Volume in LVES. All metrics were compared using paired t-tests (GraphPad Prism, Boston, MA) with significance defined as p<0.05 and Bland-Altman analysis.

Scar size analysis of conventional 2D and AutoCMR DE data in all ten pigs was performed using Segment^53^ (Medviso, Lund, Sweden). AutoCMR data was reformatted into the short-axis plane prior to segmentation. The LV was manually segmented, and infarct quantification was performed using the expectation maximization, weighted intensity, a priori information (EWA) algorithm^54^ with manual correction as needed. The resulting percent scar for 2DCMR, ACMR and TTC staining were compared using One-Way ANOVA with Dunnett’s Multiple Comparisons test (GraphPad Prism, Boston, MA). The percent scar quantified using AutoCMR was also individually compared with TTC staining and 2DCMR using intra-class correlation (ICC), assuming fixed raters rate each target^55^, and Bland-Altman analysis.

### AutoCMR at an Academic Hospital

We enrolled 179 patients to participate in AutoCMR at an Academic hospital under a Cleveland Clinic Institutional Review Board approved protocol with written consent. All participants had received a clinical CMR exam within one year prior to their AutoCMR exam.

#### Imaging

Prospective imaging was performed at the Cleveland Clinic Main Campus using a 3T Cima.X (Siemens Healthineers) and an 18-channel body coil by expert CMR technologists with >20 years of experience. Conventional 2D cine imaging was acquired in 2-chamber, 3-chamber, 4-chamber, and short-axis views (bSSFP, TR/TE=2.4/1.2ms, FA≤60°, 1.5mmx1.5mmx8mm). Gadopiclenol (Elucerim^™^) or gadobutrol (Gadavist^™^) contrast was administered at a dose of 0.1 mmol/kg, immediately followed by the proposed AutoCMR exam.

#### CMR Volume and Function Metrics

27 patients were excluded from analysis due to issues related to acquisition, reconstruction, segmentation, or 2D imaging failure. All patients included in segmentation model training (47) and validation (5) were additionally excluded from our metric analysis. AutoCMR and same-day, conventional 2DCMR measures were compared in the remaining 100 patients scanned at an Academic Hospital.

AutoCMR (ACMR) volumetric and functional parameters were compared to measures extracted from same-day, conventional 2D cine (2DCMR) images. 2DCMR was semi-automatically segmented in CVI42 (Circle Cardiovascular Imaging) and edited by a cardiology fellow. The following parameters were extracted and compared using ICC (assuming fixed raters rate each target^55^) and Bland-Altman analysis: LVED, LVES, LVEF, LVCO, LVM, RVED, RVES, RVEF, RVCO, Maximum LA Volume, Minimum LA Volume, LA EF, Maximum RA Volume, Minimum RA Volume, and RA EF.

Because atrial volumes reported by 2DCMR are derived from single-slice 4-chamber images, they are subject to errors based on slice positioning and orientation. Therefore, atrial areas (Maximum LA Area, Minimum LA Area, Maximum RA Area, Minimum RA Area) were additionally extracted from both 2DCMR 4-chamber cine images and AutoCMR cine images reformatted into 4-chamber views. As a functional measure, left and right atrial fractional area change (FAC) were calculated, where FAC = (Maximum Area – Minimum Area) / Maximum Area. Atrial area measures were compared using ICC and Bland-Altman analysis.

#### Validation Metrics

ECG signals extracted from AutoCMR raw data in the 100-patient subgroup of participants scanned at an Academic hospital were used to calculate heart rate during scanning with cubic spline interpolation and peak detection. Average heart rate and the number of detected peaks determined from ECG data and AutoCMR self-gating (SG) were compared using ICC and Bland-Altman analysis (Extended Data Figure S2).

Further validation of AutoCMR self-gating and imaging function was performed by comparing single slice endocardial segmentations in the short-axis view to same-day 2DCMR in the 100-patient subgroup. Both 2DCMR and slice matched AutoCMR images reformatted into the short axis plane were manually segmented at a single mid-ventricular slice. Manual segmentations were chosen to ensure that drawn borders were a representation of image function and not auto-segmentation performance. Single slice metrics were chosen to avoid confounding differences that arise when comparing 2D and 3D data. Specifically, the LV volumes reported by 2DCMR and calculated using a stack of thick-slice, 2D images with gaps between slices depend on the choice of the basal-most slice as well as the positioning and orientation of the prescribed short axis stack, whereas AutoCMR volumes are calculated in true 3D. Using 2D epicardial segmentations, LV area in LVED and fractional area change (FAC) were compared using ICC and Bland-Altman analysis (Extended Data Figure S2).

Vector norms perpendicular to prescribed view planes were calculated for the automatically generated 2-chamber, 4-chamber, and short-axis views of AutoCMR data and the corresponding, expert prescribed views for same-day 2DCMR images for the 100 patient subset. The angular differences between the automated and expert prescribed vector norms were calculated and analyzed with box-and-whisker plots (Extended Data Figure S5p).

### AutoCMR at a Community Health Center

We enrolled 35 patients to participate in AutoCMR at a Community Health Center under a Cleveland Clinic Institutional Review Board approved protocol. All patients had received a clinical CMR exam within one year prior to their AutoCMR exam.

#### Imaging

Prospective imaging was performed at the Cleveland Clinic Beachwood Family Health and Surgery Center using a 1.5T Altea (Siemens Healthineers) and an 12-channel body coil by CMR technologists with no CMR training. Gadopiclenol (Elucerim^™^) or gadobutrol (Gadavist^™^) contrast was administered at a dose of 0.1 mmol/kg, immediately followed by the proposed AutoCMR exam for systems with limited gradient hardware.

#### CMR Volume and Function Metrics

5 patients datasets were used for sequence development and 5 patients excluded from analysis due to issues related to acquisition, reconstruction, segmentation, or 2D imaging failure. For the remaining 25 patients, AutoCMR (ACMR) volumetric and functional parameters were compared to measures extracted from clinical 2D cine (2DCMR) images. 2DCMR values were obtained from retrospectively accessed clinical reports. The following parameters were compared using ICC (assuming fixed raters rate each target^55^) and Bland-Altman analysis: LVED, LVES, LVEF, LVCO, LVM, RVED, RVES, RVEF, RVCO, Maximum LA Area, Minimum LA Area, LA FAC, Maximum LA Volume, Minimum LA Volume, LA EF, Maximum LA Area, Minimum LA Area, LA FAC, Maximum RA Volume, Minimum RA Volume, and RA EF, where atrial areas and FAC were again calculated from single-slice AutoCMR images reformatted into 4-chamber views.

### Diagnostic and Image Quality Review

For a subset of 30 patients scanned at an academic hospital and 8 patients scanned at a community health center, AutoCMR images and conventional 2D images from a prior clinical CMR exam were reviewed by two level 3 CMR readers. AutoCMR images were provided in conventional 2D views (2-chamber, 4-chamber, short axis) and provided as complete 4D datasets for review using 3D Slicer. Readers were additionally provided age and sex for each patient but were not given a clinical history. All studies were randomized and anonymized, with AutoCMR and clinical 2D CMR images provided separately and approximately 4 weeks apart. Readers noted significant cine findings (LV aneurysm, myocardial thinning, cardiac dilation, LV or RV hypertrophy, wall motion abnormalities, aortic dilation, valve dysfunction, LV or RV dysfunction, no significant findings, other) and significant DE findings (LV late gadolinium enhancement (LGE), Pericardial LGE, cardiac mass thrombus, no LGE). Average accuracy across the two readers when compared to the clinical report was calculated and compared using McNemar’s test with significance as p<0.05. In addition, readers provided a 0–4 ranking (0: Severe, 1: Considerable, 2: Little, 3: Hardly Any) of cine and delayed enhancement image quality in the following categories: noise, susceptibility, wrap, breathing artifact, cardiac artifact. Overall image quality was calculated as an average over all categories.

### Physical and Digital Twins

#### 3D Printing

AutoCMR segmentations were imported into 3D Slicer^36,37^, where the surface was merged into a single entity and uniformly hollowed out to a consistent thickness of 3 mm before being exported as an STL file. Meshmixer (Autodesk) software was used to create an inlet in the aorta for the resin to drain, then PreForm (Formlabs) software was used to prepare for 3D printing. Surface supports and internal supports were generated with a touchpoint density of 0.55 mm and a touchpoint size of 0.4 mm for ease of removal. The part was sliced at 0.05 mm and printed on the FormLabs SLA 3B+ printer using clear resin v4 at a layer resolution of 50 μm. Printing time at full scale varied from 20 to 27 hours, depending on size over the cardiac cycle. The build platform containing the printed part was placed in Form Wash and agitated for 17 minutes in 99% isopropyl alcohol (IPA) to clean any uncured resin, then washed in clean IPA solution for another 2 minutes to remove residual particles and left to air dry for 10 minutes. Finally, it was post-cured in Form Cure at 60 °C for 17 minutes to improve its strength.

#### XR

All extended reality (XR) visualizations were performed using the software package CWRUXR and AutoCMR data saved in nifti and OBJ formats. This platform is similar to a web browser that can display source image, volume, and 3D model information so that multiple viewers can interact with the full 3D information simultaneously across a range of XR-enabled devices. Included in CWRUXR is a GPU-based raymarching shader renders 3D volumes directly in real-world scale by sampling a 3D texture in object space. The volume and raymarching rays are spatially collocated with conventional 2D slice renderings, 3D image volumes, or 3D meshes, allowing data sources to be displayed together in the same anatomical context. In this study, both a HoloLens (Microsoft, Redmond, WA, USA) and an Android-compatible phone were used to visualize the AutoCMR data.

#### FSI

A time-resolved series of registered LV surface meshes was created from AutoCMR data using 3D Slicer^36,37^. The 1-way FSI simulation was conducted using Abaqus/Explicit 2022 (Dassault Systèmes, Vélizy-Villacoublay, France) for the structural mechanics and XFlow 2022x (Dassault Systèmes) for the fluid domain, coupled via co-simulation.

In the structural domain, the LV geometry at end-diastole was meshed with linear tetrahedral elements. Dirichlet boundary conditions were prescribed on the entire endocardial surface. Fixed boundary conditions were applied at the mitral and aortic annuli to anchor the base of the ventricle. Time-varying displacement boundary conditions were imposed on all remaining nodes of the endocardial surface, derived directly from the registered mesh displacements, thereby prescribing cardiac motion throughout the cardiac cycle.

In the fluid domain, XFlow simulated LV hemodynamics using the Lattice Boltzmann method (LBM) on a fixed Cartesian mesh. Turbulence was modeled using the Smagorinsky model for improved near-wall accuracy. Blood was treated as an incompressible Newtonian fluid with a density of 1050 kg/m^3^ and a dynamic viscosity of 0.0035 Pa·s.

The LV cavity was initialized with a uniform particle distribution. Inlet and outlet boundary conditions were applied at the mitral annulus and aortic orifice, respectively. These were defined by time-resolved flow rates, calculated as the temporal derivative of LV volume derived from the registered mesh sequence.

Mesh resolution in XFlow was set to 0.8 mm globally, with adaptive refinement to 0.2 mm in regions near the LV wall to resolve boundary layer effects. Simulations were conducted over a full cardiac cycle (i.e. 0.75 s) with a fixed time step of Δt = 4 E-5 s to ensure numerical stability.

#### FEA

A validated zero-dimensional (0D) time-varying elastance circulatory model was coupled with a detailed three-dimensional (3D) finite element (FE) simulation of the left ventricle in Abaqus (Dassault Systèmes). The 0D model, calibrated using AutoCMR-derived chamber volumes and clinical cuff pressure data, provided physiological estimates of cardiac pressures and elastances, which were used as boundary conditions for the 3D model. Passive tissue mechanics followed the Holzapfel–Ogden law^56^ and an active contraction model implemented via a custom VUMAT subroutine^57^ using rule-based fiber architecture^58^. Chamber compliance and resistance were represented using fluid cavity and damping elements^59^.

Understanding the unloaded (zero-pressure) geometry of the left ventricle (LV) is essential for accurate computational modeling of patient-specific heart function under physiologic loads^60,61^. Extended Data Figure S9 compares two complementary strategies for determining the unloaded geometry. In Method I, an early-diastolic frame was identified using the high temporal resolution enabled by AutoCMR whose volume approximates the predicted zero-pressure state. This high temporal resolution offers a direct, low computational cost surrogate to iterative unloading^62–64^ based on Bols et al. (Method II) due to the lower number of iterations necessary to converge to a zero-pressure state.

## Supplementary Material

Supplement 1

## Data Availability

The main data generated or analyzed during this study are included in this article and the Extended Data. Sample raw data and 4D images are hosted on Github (https://github.com/daniellekara/AutoCMR) and will be made available upon acceptance of this manuscript. All other relevant data of this study are available from the corresponding author on request.
