# Supplementary material for: Democratizing cardiac imaging with an automated magnetic resonance exam
Source: Res Sq. 2025 Jul 18:rs.3.rs-6857034. Preprint. [Version 1] doi: 10.21203/rs.3.rs-6857034/v1 (PMC12288535; doi:10.21203/rs.3.rs-6857034/v1)
Supplement: Supplement 1 [file NIHPPrs6857034v1-supplement-1.pdf]

Supplementary Files

This is a list of supplementary files associated with this preprint. Click to download.

- SupplementaryInformation.docx
- ExtendedDataFigure.docx
- FigS1ClicksScanTime.tif
- FigS2PhantomStudy.tif
- FigureS4video.mp4
- FigS5SGvalidation.tif
- FigS6SegmentationViewFinding.tif
- FigS7AdditionalMetrics.tif
- VideoS8Hololens.mp4
- FigS9DigitalTwins.tif
- FigS10SpecialCases.tif
